# Undergraduate nursing students’ experience of clinical supervision

**DOI:** 10.4102/curationis.v41i1.1833

**Published:** 2018-11-12

**Authors:** Gabieba Donough, Marianna van der Heever

**Affiliations:** 1 School of Nursing, University of the Western Cape, South Africa; 2 Department of Interdisciplinary Health Sciences, Stellenbosch University, South Africa

## Abstract

**Background:**

Clinical supervision plays a major role in the undergraduate nursing programme. There have been some local studies addressing clinical supervision; however, there still remains a lack of knowledge and understanding how to improve quality supervision of undergraduate nursing students. This article is based on the authors’ original thesis.

**Objectives:**

The objective of the study was to explore the experiences of undergraduate nursing students on clinical supervision.

**Method:**

A descriptive design with a qualitative approach using focus group interviews was used to explore the experiences of undergraduate nursing students regarding clinical supervision. Purposive sampling was used for selection of 36 participants. The participants needed to be enrolled at the institution under study, at the time of the study, as undergraduate nursing students in order to meet the inclusion criteria. The participants also needed to have worked at clinical facilities where they received clinical supervision. Nine (*n* = 9) students were purposively selected from each year level to participate in focus group interviews. The interviews were analysed using content analysis.

**Results:**

The findings indicated both positive and negative experiences regarding clinical supervision. The results were tabulated in which the positive experiences were grouped together and were separated from the negative experiences of the undergraduate students. Positive experiences included the support that was received from supervisors. These were, however, overshadowed by many comments on negative experiences concerning the behaviour and competencies of the supervisors. The findings also confirmed that the students experienced differences in the clinical procedures demonstrated by various supervisors. Negative experiences that relate to abusive behaviour such as misuse of power were also found, as well the incongruence amongst clinical supervisors regarding clinical procedures. Recommendations were proposed to enhance clinical supervision and the learning experiences of student nurses.

**Conclusion:**

The study findings suggest a need for continuous professional development for clinical supervisors by means of in-service training as well as to maintain congruence by clinical supervisors when demonstrating clinical procedures.

## Introduction and background

Clinical supervision is widely used as a formal process of professional support for undergraduate nursing students (Franklin 2013:34) and assists with the development of professional competence and confidence amongst nursing students, ultimately enhancing the provision of quality patient care (Muthathi, Thurling & Armstrong 2017:2). The support provided through clinical supervision helps the students to link theoretical classroom knowledge with patient care in the clinical environment (Amsrud, Lyberg & Severinsson 2015:88; Brunero & Stein-Parbury 2011:87; Franklin 2013:34). Although there is evidence of positive benefits to the use of clinical supervision, there are ongoing concerns about the differences amongst clinical supervisors pertaining to the demonstration and assessment of clinical procedures (Nabolsia et al. 2012:5853). At the institution under study, the undergraduate nursing students have expressed that the various supervisors demonstrated the same clinical procedures differently. These differences tend to negatively affect how well the student learns in addition to completing their final examination successfully (Nxumalo 2011:1).

According to Regulation 425 of 2012 of the South African Nursing Council (SANC), each baccalaureate undergraduate student ought to be supervised for half an hour every week (SANC 2012). In accordance to this regulation, the institution under study employs clinical supervisors to oversee undergraduate nursing students in the clinical education environment. Clinical supervision therefore strengthens the clinical education environment because the clinical supervisors monitor and directly oversee nursing students. Lecturers at the institution also have clinical supervisory duties and assist those appointed as clinical supervisors with supervisory duties. All clinical supervisors demonstrate practical procedures to students at the institution’s skills laboratory. The clinical supervisor to student ratio at the institution under study ranges from one supervisor to 35 students and the ratio of lecturer to student ranged from one lecturer to two to three students at the time of the study.

Clinical supervision is beneficial to the students; firstly, by directly overseeing students in the clinical facility, a supportive relationship is built between the clinical supervisor and the student (Bifarin & Stonehouse 2017:1). The benefit of this supportive relationship is that it optimises teaching and learning (Nabolsia et al. 2012:5853). Lack of this supportive supervisor–supervisee relationship has been found to result in negative clinical learning experiences such as poor communication (Miller 2012:47). Secondly, clinical supervision strongly influences the student nurses’ professional development, identity and socialisation into the culture and norms of the nursing profession, including their choices of area of specialisation (Nabolsia et al. 2012:5849; Severinsson & Sand 2010:669). Negative clinical supervision experiences could also influence negative attitudes of the student to the profession, for example such students may have a desire to change their career and leave the profession (Matsinhe 2012:9; Rikhotso, Williams & De Wet 2014:1). Miller (2012:200) alludes to the link between a supervisor–supervisee’s supportive relationship and the quality of clinical experience gained during the supervision sessions, stating that if the professional relationship is poor, then the students do not gain the best experience they need to develop professionally.

Professional behaviour and respect are important elements of the clinical learning environment. Lekalakala-Mokgele and Caka (2015:5) explain that the student nurse needs to be treated with respect and as an equal partner in the clinical supervision relationship. As a result, nursing students should not experience any supervision that relates to abusive behaviour. Some studies suggest that it is essential that clinical supervisors be experienced and knowledgeable because student nurses have to develop their own skills through the time spent with supervisors (Brunero & Stein-Parbury 2011:87; Rikhotso et al. 2014:5). Other scholars allude to strengthening of clinical supervision in order to contribute to the student’s personal growth, for example good communication skills (Amsrud et al. 2015:94; Miller 2012:120; Nabolsia et al. 2012:5853). In light of the literature reviewed and the concerns that the students at the institution under study had, it was important to explore the experiences of students at the institution regarding clinical supervision.

## Purpose of the study

The purpose of the study was to ‘explore the experiences of undergraduate nursing students of clinical supervision’.

## Significance of the study

The significance and value of virtuous quality clinical supervision cannot be underestimated in the clinical education environment as it ensures that students are effectively supported and prepared to optimum standards. By exploring the experiences of the students, concerning clinical supervision evidence was received from the student’s perspective. The findings of this study provide evidence about some shortcomings in undergraduate clinical guidance. The provided recommendations could assist nursing education institutions (NEIs) to improve practices relating to clinical supervision.

## Definition of key concepts

### Clinical supervision

Clinical supervision is a formal process of professional support for undergraduate nursing students and aims to help the nursing student to develop in both their professional competence and confidence thereby ensuring safe and appropriate patient care (Franklin 2013:34).

### Clinical supervisor

In this study, the clinical supervisor is a professional nurse employed at the institution under study, who supervises the undergraduate nursing student in a clinical environment (Muthathi et al. 2017:1).

### Clinical education environment

The NEIs place the students at clinical facilities accredited by the SANC such as hospitals and community health centres to achieve clinical knowledge and skill (Traut 2013:6).

### Clinical skills laboratory

In this study, the clinical skills laboratory is a safe environment setting located at the institution under study where students can practise and acquire necessary clinical skills and competencies in a simulated environment (Mbombo & Bimerew 2012:4).

## Research design and methodology

### Design

A descriptive design with a qualitative approach using focus group interviews was used to explore the experiences of undergraduate nursing students concerning clinical supervision. A qualitative approach was considered suitable as it allowed exploring of differing individual experiences such as views, distresses, proclivities and the meaning that the experiences hold for each (Lobiondo-Wood & Haber 2014:111). Employing a descriptive design, on the other hand, was useful as it facilitated explicative summaries of the experiences of individuals related to the phenomena that was explored (Lambert & Lambert 2012:256).

### Population, sample and data collection

The total population consisted of *N* = 1001 undergraduate nursing students enrolled in the 4-year undergraduate programme at the institution under study (Donough & vd Heever 2014:44). Following ethical approval from the Health Research Ethical Committee at *Stellenbosch University*, as well the institution under study to conduct the study, the contact details of the undergraduate nursing students who formed the total population were acquired from the clinical coordinator at the institution under study. The clinical coordinator is liable for planning and organising the clinical courses which include the placements of students at clinical sites and services, coordination and lecture times (Kirtland Community College 2013:1).

De Vos et al. (2011:366) suggested an ideal focus group size to be between 6 and 10 participants. The sample therefore included nine students, each, from first, second, third and fourth year level to participate in focus group interviews. Consequently, 36 students were purposively selected who met the inclusion criteria: undergraduate nurses who were selected had to be enrolled at the institution at the time of the study and had to have worked at clinical facilities where they received clinical supervision. Students who completed the programme and those who were not enrolled at the institution were excluded.

To avoid the possibility of bias, data collection was directed by two fieldworkers who were qualified professional nurses with a Master’s degree in nursing, were proficient in how to conduct interviews and were not affiliated with the institution under study. This permitted the participants to voice their views without the possible stimulus of the first author who was employed as a clinical supervisor at the time of the study. Data were collected from the four focus groups by conducting interviews that varied between 40 and 90 min. The interviews were conducted on different days until data saturation was reached. No new information emerged with the last focus group which was group 4. This study was conducted at a nursing institution of higher education in the Western Cape. The interviews were conducted at a date and time, and at a venue convenient to the participants. The participants regarded the venues at the institution as convenient.

### Ethical considerations

The fieldworkers further validated that the participants understood the information leaflet about the study; that the declaration form to consent was signed and that ethical consideration was ensured such as anonymity and the right to refuse or withdraw at any given time. No real names were mentioned during the interview as participants were given alias names such as ‘participant 1’. These codes were also used to manage the transcripts of the interviews, that is, participant number 1, first year. The electronic copies of the data, that is, the recordings and the transcripts, are encrypted with codes and are only available to the first and second authors. The raw data will be destroyed after a 5-year period.

Ethical approval to conduct the study was granted by the Health Research Ethical Committee at Stellenbosch University (Ethics reference no.: S12/05/132), as well the institution under study.

### Instrumentation

A semi-structured interview guide was used that directed the interviews during the data collection process. According to De Vos et al. (2011:296), a semi-structured interview guide permits the first author to obtain multiple responses to set questions and allows for detailed responses. The interview guide consisted of a list of four open-ended questions that were based on the objective of the study. Open-ended wide-ranging questions for each interview were used such as: ‘Tell me about your experiences of clinical supervision’. Probing words for exploring the experiences on clinical supervision were: the need for supervision, the value thereof and the quality of supervision.

### Data analysis

The four focus group interviews were carried out. The interviews were transcribed verbatim to improve the trustworthiness of the data collected. The five steps described by Terre Blanche, Durrheim and Painter (2006:322) were used to analyse the content. These included: Step 1 – Familiarisation and immersion: the transcriptions were read repetitively and the recordings were listened to repetitively in order to gain a full understanding of the experiences and to become familiar with the information. Step 2 – Inducing themes: the data that were derived from the interviews were broken down, examined and compared to determine patterns, similarities and differences. Thereafter, the themes that naturally supported the data identified were grouped into specific themes and subthemes; Step 3 – Coding of transcripts was completed by identifying the essential messages in each transcript and these pieces of text were then labelled; Step 4 – Elaboration allowed the first author to explore the themes more closely and to revise the coding system. Step 5 – Interpretation and checking: each theme was re-evaluated for possible misinterpretations, whether important issues were overlooked and whether the biases of the first author might have coloured the final themes.

### Trustworthiness

The first author ensured trustworthiness of the qualitative data through the process of credibility, dependability and confirmability as advised by Lincoln and Guba (1985:290).

Credibility was ensured through member checking. Accordingly, the first author contacted each participant and provided them an opportunity to comprehend the content of the transcripts, discuss the themes and the subthemes and the interpretation thereof as advised by Brink, Van der Walt and Van Rensburg (2008:118). Verification of the data occurred when the participants were satisfied with the themes and the subthemes as well as the interpretations thereof.

Transferability, whether the findings are applicable to another context, rest with the reader. Consequently, to allow another first author the opportunity to assess whether the findings are applicable to their context, the research report provides thick descriptions of the sample and data collection and analysis processes, how the information was received, the relationship the first author had with the participants and why fieldworkers were used to collect the data (De Vos et al. 2011:346).

To ensure dependability, the 1st author verified the authenticity of the recordings of the interviews that were completed by the fieldworkers against the transcripts. The supervisor involved in the study verified the relationship between the transcripts of the recordings and the final themes for authentication and accuracy.

In addition, the transcribing of the audio recordings was completed by a professional audio transcriptionist who proofread all the documents (Donough & vd Heever 2014:31).

## Findings

The findings are presented in Table 1 and emphasise the themes and subthemes that arose from the information.

**TABLE 1:** Themes and subthemes.

Experiences	Themes	Subthemes
Positive experiences	Support	Alleviation of student’s feelings of fear
Negative experiences	Professionalism	Appointment management Incompetence of the supervisor
	Duties to uphold supervision	No show of clinical supervisors Focus on administration versus student guidance Assessment versus developmental focus
	Experiences that relate to abusive behaviour	Misuse of power
	Clinical supervision process	Incongruence amongst clinical supervisors Attention to private instead of professional matters, for example private calls Short formative assessment procedures

Five themes and 10 subthemes emerged from the interviews: support, professionalism, duties to uphold supervision, experiences that relate to abusive behaviour and the clinical supervision process. The 10 subthemes that emerged from the five major themes are displayed in Table 1.

### Theme 1: Support

First-year students specifically experienced fear for the unknown clinical environment. They therefore regarded the presence of clinical supervisors as supportive.

#### Subtheme 1: Alleviation of student’s feelings of fear

The physical presence of supervisors seemed to have alleviated feelings of fear.

‘my experiences with the clinical supervision was a good experience, because you know, as we go into the field as the first years, we have that fear of not doing good in the hospitals, but with them assisting us and being there next to us helps us a lot.’ (Group 1, first year, Participant 1)

In addition, students experienced the availability and guidance of clinical supervisors as reassuring because some staff employed at the clinical facilities were apparently unwillingly to assist the student nurses because of remuneration issues.

‘hospitals the staff would say “why don’t your supervisors teach you guys these things, why are you coming to us for teach, we must teach you, we don’t get paid for this, your facility needs to teach you”.’ (Group 4, fourth year, Participant 7)

### Theme 2: Professionalism

Some students had negative experiences relating to the professional behaviour exhibited by clinical supervisors such as not being punctual and apparent incompetence.

### Subtheme 1: Appointment management

Participants verbalised deficits in terms of communication and consideration between the supervisor and the student. These related to incidences where supervisors would arrive late for a scheduled clinical evaluation. In instances where the supervisors were not punctual, the actual time that the student was released from clinical duties was prolonged, ultimately ruining the image of the student and that of the institution of higher education where the student was enrolled.

‘You wait an hour to an hour and a half for your supervisor to come, then you have your supervisor you’re busy for two hours and you come back to the ward and immediately the sister that’s in charge she thinks that you’re walking around.’ (Group 2, second year, Participant 3)

#### Subtheme 2: Incompetence of the supervisor

Some students related incidences where supervisors were not able to execute certain procedures requested by the students. These incidences create the idea that some clinical supervisors might lack experience and the need for continuous professional development of supervisors. The subtheme, incompetence of the supervisor, was prevailing amongst the third-year group.

‘when we ask the supervisors to demonstrate how to perform, how to demonstrate like mechanisms of labour. To be honest I actually realized that, okay I didn’t ask all of them but the ones that we asked, they don’t know how to demonstrate it.’ (Group 3, third year, Participant 3)

‘Then I got her for the reev [*re-evaluation*] so I asked her what happened? Where did I went wrong? And she looked and said no you didn’t mention about the Trendelenberg position. So I asked her what is this position, to my surprise she didn’t even know what was the Trendelenberg position.’ (Group 3, third year, Participant 1)

### Theme 3: Duties to uphold supervision

The third theme concerns the duties of the clinical supervisor during clinical supervision as experienced by student nurses.

#### Subtheme 1: No show of clinical supervisors

Some participants explained that, at times, they did not receive any clinical supervision and that some supervisors are known for being absent for clinical supervisory duties.

‘we know some of them [*supervisors*] are not going to pitch.’ (Group 3, third year, Participant 6)

‘from last semester … there’s no one [*supervisor*] came to see us and how we doing.’ (Group 1, first year, Participant 6)

However, when students do receive supervision, they described their experiences of the actual time spent on individual supervision as insufficient. Reasons for this insufficient supervisory time were postulated to be the dual roles of lecturers, as well as the high supervisor–student ratio. This is supported by the following quote:

‘Like some will just go and five minutes and they’ll be gone.’ (Group 4, fourth year, Participant 1)

‘They don’t spend enough time with us so that we can have to ask them questions.’ (Group 3, third year, Participant 1)

‘supervisors who are lecturers, that’s a problem for me because that’s what I noticed because she’s a lecturer and she’s a supervisor and she doesn’t have time and she will tell you that ‘I don’t have time.’ (Group 2, second year, Participant 6)

#### Subtheme 2: Focus on administration versus student guidance

Some participants described that clinical accompaniment seemed to reflect an administrative nature as the supervisors would complete documentation related to clinical supervision only and that no accompaniment happened.

‘They come to the facility, they sign forms and the register and then off they go.’ (Group 3, third year, Participant 2)

‘So I feel that they shouldn’t be there only with you just to make you sign a piece of paper to say they’ve been there with you for five minutes.’ (Group 4, fourth year, Participant 1)

#### Subtheme 3: Duties to uphold supervision – Assessment versus developmental focus

Other students experienced that accompaniment would only relate to the completion of compulsory assessment procedures instead of providing clinical guidance. Some students started to accept that assessment procedures were more important than clinical guidance. Therefore, students perceived the clinical supervisor as assessment-orientated.

‘I wish they would like not only come when we have an assessment to do … they only pitch when there’s something that you have to do … the only things I make sure I know is the vital signs, the wound care and that’s it. But the other stuff it’s like they’re not important because we’re not frequently showed how to do them, so I wish they would come when other days.’ (Group 1, first year, Participant 2)

### Theme 4: Experiences that relate to abusive behaviour

Other negative experiences of student nurses related to abusive behaviour exhibited by the clinical supervisors, such as the misuse of power.

#### Subtheme 1: Misuse of power

Some clinical supervisors apparently have a tendency to misuse the power entrusted to them.

‘I feel that the supervisors use the fact that they’re authority figure, I think they misuse it sometimes.’ (Group 4, fourth year, Participant 9)

‘I’ve also experienced like they [*clinical supervisor*], you know, on a power trip, like they’re dominating you.’ (Group 2, second year, Participant 4)

The misuse of power translated in threatening students with deliberate failure of clinical assessments. For example, some supervisors would not allow the students to voice their opinion or question low marks, indicating that they have failed a procedure. These are illustrated in the following comments:

‘I’m [*clinical supervisor*] going to enjoy marking you because I must make sure that I also go deeper into my knowledge in order to mark you down … He actually said it.’ (Group 3, third year, Participant 3)

‘they [*supervisors*] will even discuss with their other colleagues, “watch out for that one, watch out for that one” and you’ll be marked [*in the clinical exam*].’ (Group 4, fourth year, Participant 8)

Misuse of power also transpired through verbal abuse displayed towards student nurses by clinical supervisors in the presence of other clinical staff. Consequently, some students perceived certain clinical supervisors as unprofessional and rude.

‘she’s going on like a crazy woman to the unit manager’s office and coming back to me, shouting, shouting, shouting and I’m following her like a puppy.’ (Group 4, fourth year, Participant 7)

### Theme 5: Clinical supervision process

The clinical supervision process seemed to contain questionable practices such as incongruence amongst clinical supervisors, attention to private instead of professional matters, for example private calls, and short formative assessment procedures.

#### Subtheme 1: Incongruence amongst clinical supervisors

The institution under study has a practical workbook reflecting guidelines for the completion of clinical procedures. Various participants experienced that supervisors would demonstrate the same procedure differently, irrespective of whether demonstrations were provided at the different clinical facilities where students are placed or at the institutions clinical skills laboratory. It therefore appears that the guidelines contained in the practical workbook are perhaps not always considered, eventually creating confusion that might impact the throughput of the students because the guidelines serve as assessment criteria against which students are evaluated.

‘every a supervisor has her way of doing things, of teaching us, so every time when you get a new supervisor you have to adapt on how maybe she wants … to do certain things, certain way.’ (Group 1, first year, Participant 3)

‘So the one supervisor in the lab they will show you, this is the procedure, this is the way … but when you’re in the clinical facility, when I did my demonstration when I practice it, the supervisor was saying “no, you’re doing it wrong”.’ (Group 4, fourth year, Participant 1)

#### Subtheme 2: Attention to private instead of professional matters, for example private calls

From time to time, clinical supervisors apparently answered private calls while students were performing clinical procedures for assessment purposes. Students reported to have experienced the calls as distracting. At times, some supervisors would exit the room where the assessment happened because of calls. Some students explained that they were confused as to why they had failed because the supervisor left the room.

‘During assessments they tend to be on their cell phones, doing whatever and it’s a bit distracting because she’s supposed to be, or he’s supposed to be focusing on you.’ (Group 3, third year, Participant 6)

‘then I officially started with my procedure then my clinical supervisors cell phone rang and she left … she gave me a failed mark.’ (Group 4, fourth year, Participant 8)

#### Subtheme 3: Clinical supervision process – Short formative assessment procedures

At times, some clinical supervisors would be receptive to shortened clinical formative assessment procedures. Some participants reported that they had received inflated marks for these procedures that were carried out in an unusual short time. Although they were happy with the inflated marks, they also realised that they might not be fully prepared for the final summative clinical examination because the clinical supervisor only pointed out minimal mistakes during these shortened formative clinical assessments.

‘how can a supervisor do a procedure for you, an emergency procedure, emergency training procedure for five minutes and then you’re done.’ (Group 2, second year, Participant 6)

‘for us we’re just happy that we’re getting marks and all that but it doesn’t help us at the end … she will give you hundred percent, okay that’s fine, but it doesn’t help you.’ (Group 2, second year, Participant 6)

## Discussion

Various research findings (Lindgren et al. 2005:822; Tomlinson 2015:1) confirm that students experience clinical accompaniment as supportive and that the support contributes to the lessening of fear and anxiety experienced in the clinical setting (Muthathi et al. 2017:6). The authors, Muthathi et al. (2017:6), reported that support provided to the students’ contributed to the ultimate progress of the students enrolled in nursing programmes.

Irrespective of support contained in clinical supervision, professional behaviour displayed by clinical supervisors enhances the quality of clinical supervision. Professionalism also relates to the skills, competence and behaviours (Thistlethwaite & McKimm 2015:85) of the clinical supervisor. However, the attainment of professionalism is ongoing (Morris & Faulk 2014:96). Professionalism relates to being punctual and that one should demonstrate accountability and responsibility (Mellish, Brink & Paton 2009:10). However, research findings reported at the International Scientific Conference in 2012 revealed that because of the rise in student numbers, clinical supervisors have limited contact sessions with students in the clinical setting (XL Millennium Conferences 2012:1). Preferably, the ratio of supervisor to supervisees should be 1:8 (Lynch et al. 2009:80). Conversely, Schellenberg (2012:485) recommended a ratio of 1:6. Similarly, Maart (2011:13) recommended a ratio of 1:6/7. However, the National Department of Health recommended a ratio of 1:15 (National Department of Health 2013:86). Ultimately, each supervisor should supervise a manageable number of supervisees (West London Mental Health NHS Trust 2011:6). At the institution under study, the ratio of clinical supervisor to student ranged from one supervisor to 35 students at the time of the study. These high ratios might therefore implicate issues related to punctuality and the provision of support to all.

Directives of the Royal College of Nursing (2012:1) advise that every student should have access to clinical supervision and each supervisor should supervise a recommendable amount of students. Large student ratio per supervisor results in limited contact sessions with students (Jeggels, Traut & Africa 2013:1). Having a manageable ratio will also ensure optimal student learning (National Department of Health 2013:95). Similarly, Regulation 425 of the SANC that reflects standards for the baccalaureate level stipulates that each student must be supervised at least half an hour every week (SANC 2012:1; South African Qualifications Authority 2010:4). It is therefore beneficial that the time spent during clinical accompaniment be meaningful to enhance the supervisor/supervisee experience. However, the findings of the current study signify the possibility that the high supervisor/supervisee ratio, that is, 1:35, might implicate the quality of clinical supervision because quality clinical supervision is related to the availability and utilisation of qualified supervisors (Jonsén, Melender & Hilli 2013:260–262). Although a signed document verifies that the student was indeed followed up, the actual bedside teachings and guidance did not occur. Consequently, the perceived gaps in the supervisory system led students to have a compromised image of clinical supervision.

In addition, the findings of the current study revealed that actual accompaniment was often replaced with the completion of compulsory assessments, suggesting the need for supervisors to improvise because of time constraints in order to complete their duties. As student learning is also dependent on sufficient time spent between the student and supervisor through face-to-face meetings on a regular basis (University of South Africa 2012:5), these inconsistencies relating to accompaniment suggest the possibility that student learning be compromised because of time deficiencies.

Notwithstanding time constraints, Sosteric (2012:1) relates that educators are powerful representatives who display shameful and abusive behaviour towards students. The findings of a study completed by Lekalakala-Mokgele and Caka (2015:5) also confirmed the abuse of power amongst lecturers and that abuse transpired through verbal abuse such as shouting and threatening students with possible failure. In this respect, reprimanding or scolding a student in front of their peers or an audience is wrong (Whitaker & Breaux 2014:108). Ideally, the reprimanding of students should be done in private and in a professional manner (Reinke, Herman & Stormont 2013:41).

Other best practices regarding clinical supervision include consistency in performing procedures (Muthathi et al. 2017:5) and incongruence amongst the clinical supervisors pertaining to the execution of clinical procedures is contradictory to best practices. Muthathi et al. (2017:5) advise that the demonstration of procedures be standardised. Although the finer detail of the procedures carried out by supervisors could differ, all clinical supervisors should use the same guidelines for clinical demonstrations and assessments.

The findings of the current study also revealed the display of unprofessional behaviour by supervisors and that role modelling of professionalism is lacking. It is, however, required that professional nurses serve as role models of professionalism because role modelling is a technique that permits students to acquire new behaviours by imitating proficient behaviour (Ahanonu & Waggie 2015:1).

## Conclusion

The undergraduate nursing students’ experiences on clinical supervision were explored to obtain information from the students’ viewpoint. The finding indicated that undergraduate nursing students at the institution under study had both positive and negative experiences regarding clinical supervision.

However, the negative experiences in the clinical field, encountered by most participants amongst all the groups, overshadowed the positive experiences mentioned by some participants.

Recommendations were therefore proposed based on the findings of the study to improve the quality of clinical supervision and the learning experiences of student nurses.

## Recommendations

The following recommendations were developed based on the findings of this study:

- An induction programme at the institution under study with peer and formal evaluation would benefit the newly appointed clinical supervisors, which is intended to prepare the new supervisor for their specific role. This can be performed by the clinical coordinator at the institution. The induction programme will contribute to the high calibre of clinical supervisor which will contribute to the quality in clinical supervision.
- Clinical supervisors should attend in-service training and continuous professional development in the form of educative clinical workshops to enhance their clinical knowledge and performances. The aim is to revise clinical nursing procedures and to share knowledge between clinical supervisors. This intervention will ensure the implementation of evidence-based practices and the standardisation and congruence of procedures. Additionally, it will contribute to improved competence, skills development, better staff morale and a motivated workforce. Workshops can also include discussing professionalism and ethics aiming to guide clinical supervisors on professionalism and role modelling. This can also be performed at the faculty in the institution under study. Through these in-service training and workshops, the quality and revitalisation of clinical supervision at the institution under study will be strengthened.
- The large student ratio per supervisor resulted in limited contact sessions with students and clinical supervisors arriving late for scheduled appointments and also led to frustrations amongst clinical supervisors. It is therefore recommended that the ratio of students per clinical supervisor needs to be manageable with a ratio of 1:15 according to National Department of Health (2013). Additionally, employing more clinical supervisors can also help reduce the large student ratio per supervisor.
- Support is definitely needed for clinical supervisors such as debriefing sessions and peer counselling to lessen the clinical supervisors’ stress and work pressure. These sessions can be conducted on a quarterly basis per year level with the clinical coordinator and all staff involved in clinical supervision at the institution under study.

## Limitations

The study only focussed on students at one undergraduate nursing institution and omitted the broader populace of undergraduate nursing at other institutions. In addition, the experiences of the supervisors and information from their view were also not explored. Therefore, future research exploring the viewpoint from the supervisor at the institution under study could be beneficial.
